# The Clinical and Cost-Effectiveness of 4 Enzyme-Linked Immunosorbent Assay Kits for Monitoring Infliximab in Crohn Disease Patients: Protocol for a Validation Study

**DOI:** 10.2196/11218

**Published:** 2018-10-19

**Authors:** Thomas Langford, Zehra Arkir, Anastasia Chalkidou, Kate Goddard, Lamprini Kaftantzi, Mark Samaan, Peter Irving

**Affiliations:** 1 King's Technology Evaluation Centre School of Biomedical Engineering and Imaging Sciences King's College London London United Kingdom; 2 Reference Chemistry Laboratory Viapath St Thomas' Hospital London United Kingdom; 3 Department of Gastroenterology Guy's & St Thomas' National Health Service Trust London United Kingdom

**Keywords:** antidrug antibodies, anti-TNF, Crohn’s disease, ELISA, inflammatory bowel disease, infliximab, therapeutic drug monitoring

## Abstract

**Background:**

Currently, treatment decisions for people with Crohn disease are based on clinical judgment and trial and error. Consequently, people may continue to receive high drug dosages and experience unnecessary toxicity when it is possible to reduce or discontinue without a detrimental effect on clinical outcomes. Therapeutic drug monitoring (TDM) involves regularly testing blood samples for drug and antibody levels that could help clinicians identify the optimal treatment strategy and pre-empt treatment failure. However, heterogeneity in the assays can lead to a discrepancy in results and difficulties in decision-making. Standardization of the kits, and therefore results, would allow clinicians to optimize the use of biologics. Currently, there is also a lack of evidence for the cost-effectiveness of TDM using commercial test kits.

**Objective:**

This study aims to analyze the clinical and cost-effectiveness of 4 commercial enzyme-linked immunosorbent assay (ELISA) kits (LISA TRACKER, IDKmonitor, Promonitor, and RIDASCREEN) to generate evidence which could support a recommendation for wider adoption in the National Health Service.

**Methods:**

We propose to carry out a prospective-retrospective predictive biomarker validation study using the blood samples and clinical/utilization data collected during the ongoing SPARE trial (NCT02177071). A total of 200 stored samples from people with Crohn's disease who respond to treatment with infliximab will be used along with clinical and cost data from the trial. We will investigate the relationship between the drug and antidrug antibody levels with the main clinical outcomes (relapse rate at 2 years and time spent in remission), as well as resource utilization and quality of life.

**Results:**

Funding is being sought to conduct this research.

**Conclusions:**

This is the first study to compare the 4 ELISA kits for monitoring infliximab in patients with Crohn disease. It aims to address the uncertainties in the potential benefits of using the technologies for TDM.

**International Registered Report Identifier (IRRID):**

PRR1-10.2196/11218

## Introduction

### Background

Crohn disease and ulcerative colitis are the main conditions described as inflammatory bowel disease. Crohn disease is a chronic, fluctuating inflammatory condition of the digestive tract that can affect both adults and children. The main symptoms include chronic or nocturnal diarrhea, abdominal pain, rectal bleeding, and weight loss. The disease follows an unpredictable relapse (active disease) and remission (no symptoms) course with significant variation in the pattern and complexity of symptoms. During a relapse, patients often suffer substantial morbidity and require intensive treatment, including invasive investigations, costly drugs, and surgery. The prevalence of inflammatory bowel disease in the United Kingdom (UK) is estimated to be 240,000 with Crohn disease affecting about 115,000 people [1]. In 2006, the cost of inflammatory bowel disease to the National Health Service (NHS) was estimated at £720 million, based on the prevalence and an average cost of £3,000 per patient per year [2]. The cost today is likely to be significantly higher with the availability of new biological therapies that have an average annual cost per patient estimated between £10,000 and £15,000 [3]. At Guy’s and St Thomas’ NHS Foundation Trust, the Inflammatory Bowel Disease Service has approximately 600 patients receiving biological therapies incurring costs of £6 million per year.

Currently, there is no cure for this lifelong condition. Drugs are used to suppress the overactive immune system in people with Crohn disease, with the intention of inducing and maintaining remission. However, 30% of patients fail to respond to first-line drugs and will then be considered for anti-tumor necrosis factor (TNF) alpha biological therapies, such as infliximab (IFX) and adalimumab (ADAL). Anti-TNF alpha treatment aims to induce remission and prevent relapse by targeting the inflammation-causing protein, TNF alpha, rather than suppressing the immune system as a whole. Despite this, loss of response and relapse are common. The annual risk of loss of response is estimated at 13% per patient [4]. The typical response to loss of response is dose intensification; however, the underlying cause of the loss of response is not fully understood. The main hypothesis is that some patients develop antibodies against the biologics preventing the concentrations of the drug in the patient’s bloodstream from reaching levels required to maintain remission. People whose disease responds to a TNF alpha inhibitor may continue receiving the same level of the drug even when it may be possible (or even beneficial [5]) to reduce the dose or withdraw the drug entirely without any detrimental effect on clinical outcomes. This continued treatment may lead to people experiencing unnecessary side effects. Treatment decisions for people with Crohn disease are based on clinical judgment and trial and error. Measuring the levels of TNF alpha inhibitors and associated antibodies in the blood could help clinicians to identify the best treatment strategy for a person with Crohn disease. This is known as therapeutic drug monitoring (TDM).

Numerous commercial kits are available for TDM of biologics for Crohn disease. The literature is inconclusive on whether enzyme-linked immunosorbent assay (ELISA) testing improves patient outcomes or is cost-effective [6-10], as they are not routinely used to optimize treatment. The optimal approach and frequency of delivering TDM are also uncertain. In a study of health care professionals’ routine practice, only 45% reported using TDM during maintenance therapy for patients in remission [11]. One reason for this being heterogeneity in the assays which can lead to a discrepancy in results. Standardization of the kits, and therefore results, would allow clinicians to optimize the use of biologics.

In 2016, the National Institute for Health and Care Excellence (NICE) published diagnostics guidance on TDM of TNF alpha inhibitors (IFX and ADAL) in Crohn disease (referred to as DG22) [12]. DG22 evaluated the clinical and cost-effectiveness of 3 ELISA kits. The 3 commercial kits were (1) LISA TRACKER (Theradiag, Croissy-Beaubourg, France), (2) Immundiagnostik/BioHit Healthcare, Bensheim, Germany, and (3) Promonitor (Grifols Diagnostic Solutions, Emeryville, United States). They were considered for testing levels of TNF alpha inhibitors and antidrug antibodies in 2 populations: (1) people with Crohn disease whose disease responds to treatment with TNF alpha inhibitors and (2) those who experience secondary loss of response. The DG22 found many limitations within the evidence identified. No studies were found to be assessing direct clinical outcomes for any of the commercially available test kits, and there was a paucity of evidence on cost-effectiveness in general. The DG22 concluded that although the kits show promise, there is insufficient evidence to recommend routine adoption across the NHS.

In response to the uncertainties identified in DG22, NICE recommended future research focus on addressing gaps in the current evidence and investigating the potential benefits of using ELISA kits for Crohn disease treatment monitoring within the NHS. In May 2016, NICE requested King’s Technology Evaluation Centre (KiTEC), based at King’s College London, to plan and obtain funding for research that will address the uncertainties mentioned above. As part of this research, KiTEC will carry out a prospective-retrospective predictive biomarker validation study to assess the clinical and cost-effectiveness of the ELISA kits. This study will use the stored samples and the clinical and cost data from a multicenter, international randomized controlled trial “Prospective Randomized Controlled Trial Comparing Infliximab-Antimetabolites Combination Therapy to Antimetabolites Monotherapy and Infliximab Monotherapy in Crohn's Disease Patients in Sustained Steroid-Free Remission on Combination Therapy” (SPARE) NCT02177071. Results from this study would provide further impetus for the remaining NICE research recommendations:

- Assess the analytical and clinical validity of the tests and develop standardized primary reference standards
- Prospectively evaluate the clinical utility of the ELISA kits in people with Crohn disease who are losing responsiveness to infliximab

### The SPARE Clinical Trial

One of the treatment strategies used in the management of severe active Crohn disease that has not responded to conventional therapy is combination therapy. In this approach, an immunosuppressant drug (also known as an antimetabolite) such as azathioprine, mercaptopurine or methotrexate and an anti-TNF alpha agent called infliximab are used together. This combination is highly effective in inducing remission. The multinational “Study of Biologic and Immunomodulator Naïve Patients in Crohn's Disease” (SONIC) trial NCT00094458 (Europe, Israel, and North America) [13] demonstrated that infliximab plus azathioprine combination therapy was superior to infliximab monotherapy and azathioprine monotherapy to achieve steroid-free remission and mucosal healing in antimetabolites-naïve steroid-dependent or steroid-refractory patients.

Despite this superiority, maintaining such combination therapy long-term may generate cost and safety issues. The NICE and Scottish Medicines Consortium [14] mandate reassessment of patients on combination therapy at 12 monthly intervals with consideration of drug withdrawal where patients are in sustained deep remission.

However, there is currently insufficient data on relapse and recapture rates to inform such decision making [15-18]. In response to the lack of evidence, a prospective open-label, international 3-arm trial SPARE was launched in October 2015. This trial assessed the benefits of the continuation of combination therapy and the feasibility of infliximab or antimetabolites discontinuation in patients in sustained steroid-free remission after prolonged treatment with a combination of infliximab and antimetabolites. The purpose of the SPARE study, therefore, is to find the safest and most effective way for patients to discontinue their combination therapy by comparing 3 different withdrawal strategies:

- Continued combination therapy (immunosuppressant drug and infliximab)
- Immunosuppressant drug alone (so infliximab discontinued)
- Infliximab alone (so immunosuppressant discontinued)

This will help find out which strategy has the best chance of maintaining remission of Crohn disease and to determine the risk factors for (1) disease flare, (2) side effects, (3) quality of life, and (4) the impact on people’s social and professional life. The aim is to be able to identify which patients for whom discontinuation of immunosuppressant drug or infliximab could be considered after 1 year of treatment and what would be the best treatment strategy. The SPARE trial has a planned duration of 2 years main study plus 2 years follow-up. The main coprimary outcomes are (1) clinical relapse rate at 2 years and (2) mean remission duration within 2 years. Secondary outcomes include (1) time to relapse in each arm, (2) treatment failure rate, (3) time to treatment failure, and (4) tissue damage progression. The estimated completion date is January 2020.

### Other Relevant Clinical Trials

There are many additional ongoing or recently completed trials which fulfill the requirements of our validation study and could be contacted to obtain samples. Three trials were identified, 2 have taken place in Europe and 1 in America. All include populations with luminal Crohn disease treated with infliximab who have been in remission for at least 6 months (Textbox 1).

### Study Objectives

The primary objective of this study is to validate the ELISA kits by examining the relationship between infliximab and antiinfliximab antibody levels measured in duplicate. These will be compared with the main clinical outcomes (relapse rate at 2 years and mean restricted time spent in remission). The secondary objective is to evaluate the effect of monitoring infliximab and antidrug antibody concentrations in serum samples on resource utilization and health-related quality of life in patients with Crohn disease who respond to treatment with infliximab.

**Three other relevant clinical trials.**

1. **“Switching from originator infliximab to biosimilar CT-P13 compared with maintained treatment with originator infliximab” (NOR-SWITCH)**
  - Total of 155 adult patients with Crohn disease were recruited in Norway
  - Blood samples were collected and stored in a biobank [19]
  - Completed in 2017
  - NCT02148640
2. **“Precision Dosing of Infliximab Versus Conventional Dosing of Infliximab” (Precision IFX)**
  - Currently recruiting 800 adult and pediatric patients with Crohn disease or ulcerative colitis in the United States who are in remission
  - Expected to be completed in December 2018
  - NCT02624037
3. **“GIS-SUSANTI-TNF-2015” (Anti-TNF Discontinuation)**
  - Currently recruiting 300 adult participants with ulcerative colitis or Crohn disease in Spain
  - Primary completion date is December 2020
  - NCT02994836

## Methods

### Interventions

Commercial laboratories have developed various assay procedures for TNF alpha inhibitors and antibodies against TNF alpha inhibitors. The LISA TRACKER (Theradiag, Croissy-Beaubourg, France), (Immundiagnostik/BioHit Healthcare, Bensheim, Germany), Promonitor (Grifols Diagnostic Solutions, Emeryville, United States) and RIDASCREEN (R-Biopharm, Darmstadt, Germany) are particular examples of these essays classed as solid-phase ELISAs. They are intended to be used for measuring the levels of TNF alpha inhibitors and antibodies against TNF alpha inhibitors in the blood of people having treatment with biologics for Crohn disease. However, these kits vary in designs and there is a lack of standardization. Their unique features are detailed in the following (see Tables 1 and 2).

### Study Population

The study population is patients who have been in steroid-free remission for at least 6 months undergoing infliximab/ antimetabolites combination therapy scheduled for at least 1 year. The population must also have had infliximab treatment administrated every 8 weeks for the last 6 months. Detailed inclusion and exclusion criteria are presented below (Textbox 2).

**Table 1:** Drug assay kit specifications.

Details	Drug assay			
	IDKmonitor	LISA TRACKER (Infliximab)	Promonitor (Infliximab)	RIDASCREEN
Microplate coating	Anti-IFX^a^ monoclonal antibody	Recombinant TNF^b^ alpha	Anti-TNF alpha monoclonal antibody bound to recombinant TNF alpha	TNF alpha
Primary conjugate	HRP^c^-conjugated anti-IFX monoclonal antibody	Biotinylated antihuman IgG1^d^ antibody	HRP-conjugated anti-IFX monoclonal antibody	HRP-conjugated anti-IFX monoclonal antibody
Secondary conjugate	—^e^	Streptavidin-HRP conjugate	—	—
Detection method	TMB^f^	TMB	TMB	TMB
Measurement range (µg/mL)	0.4-45.0	0.3-16.0	0.035-14.4	0.1-12.0^g^

^a^IFX: infliximab.

^b^TNF: tumor necrosis factor.

^c^HRP: horseradish peroxidase.

^d^IgG1: immunoglobulin G subclass 1.

^e^TMB: tetramethylbenzidine

^f^Not applicable.

^g^(2.0-48.0 on dilution).

**Table 2:** Antidrug antibody assay kit specifications.

Details	Antidrug antibody assay			
	IDKmonitor (total)^a^	LISA TRACKER (free)	Promonitor (free)	RIDASCREEN (free)
Microplate coating	Streptavidin	IFX^b^	IFX	IFX
Primary conjugate	HRP^c^-conjugated IFX	Biotinylated-IFX	HRP-labelled IFX	Biotinylated-IFX
Secondary conjugate	Biotinylated-IFX	Streptavidin-HRP conjugate	—^d^	Streptavidin-HRP conjugate
Detection antibody	TMB^e^	TMB	TMB	TMB
Measurement range	10 arbitrary unit/mL^f^	10-200 ng/mL	2-1440 arbitrary unit/mL	2.5-125 ng/mL^g^

^a^Samples are subjected to manual infliximab-antidrug antibody dissociation step.

^b^IFX: infliximab

^c^HRP: horseradish peroxidase

^d^Not applicable.

^e^TMB: tetramethylbenzidine.

^f^Semiquantitative, single cutoff.

^g^(20-1000 ng/mL extended range).

**Study inclusion and exclusion criteria.**

**Inclusion criteria**

- Diagnosis of luminal Crohn disease
- Male or female, ≥18 years of age to ensure data is comparable with other participating regions
- Currently treated using a combination therapy of infliximab and antimetabolites for luminal Crohn disease
- Combined therapy with scheduled infliximab and antimetabolites for at least 12 months
- Scheduled administration of infliximab 5 mg/kg every 8 weeks over the last 6 months
- Antimetabolites administered at a stable dosage for the last 6 months (ie, at least 1 mg/kg or 2 mg/kg for mercaptopurine and azathioprine, respectively, or the highest tolerated dosage if intolerance to standard dose and at least 15 mg/week subcutaneously for methotrexate)
- Patients in steroid-free clinical remission for at least 6 months according to the retrospective assessment of the patients’ files
- Crohn’s Disease Activity Index <150 at baseline
- Adequate contraceptive (as judged by the principal investigator) during the whole study for female participants of childbearing potential
- Patients able to understand the information provided to them and to give written informed consent for the study

- Patients who have presented a severe acute or delayed reaction to infliximab
- Perianal fistulae as the main indication for infliximab treatment
- Active perianal/abdominal fistulae at time of inclusion, defined by active drainage
- Patients with ostomy or ileoanal pouch
- Pregnancy or planned pregnancy during the study or breastfeeding
- Inability to follow study procedures as judged by the investigator
- Noncompliant subjects
- Participation in another therapeutic study
- Steroid use ≤6 months prior to screening
- Currently receiving steroids, immunosuppressive agents (other than purine, methotrexate), biologic treatment (other than infliximab) or thalidomide

The overall SPARE target is 300 randomized patients (100 per arm) worldwide over 20 months from 70 centers. Currently, the plan of recruitment is as follows:

- 100 patients in 20 centers (France)
- 70 patients in 21 centers (UK)
- 50 patients in 10 centers (Sweden)
- 45 patients in 10 centers (Germany)
- 35 patients in 9 centers (Belgium)

The KiTEC study will focus on 2 of the 3 study arms in which participants are being treated with infliximab. Participants from the other arm were excluded because they are not currently being treated with infliximab.

### Study Design

The retrospective-prospective study will focus on patients with luminal Crohn disease who have sustained remission and are being treated with infliximab. Assays will be prospectively performed in duplicate on 200 blood serum samples. The samples were retrospectively collected from the SPARE trial. Our primary collaborator from the SPARE trial is Dr Edouard Louis (Centre Hospitalier Universitaire de Liège, Belgium), who is also the principal investigator.

The SPARE trial is an open-label, multicenter trial with 3 parallel randomized arms comparing 3 strategies of maintenance therapy in patients in sustained clinical remission without steroids for at least 6 months and having been treated by a combination of antimetabolites and infliximab for at least 1 year (Multimedia Appendix 1). Participants in study arms 1 and 3 who have sustained remission (referred to as “responders” and denoted as G2, G6, G8, and G12 in Multimedia Appendix 1) are the population focused on in this study. The SPARE trial is estimated to run for 5 years including 2 years of enrolment, 2 years of patient follow-up, and 1 year of data analysis. The trial began on October 2015 and has an estimated study completion date on January 2020. The KiTEC study is planned to last 18 months and overlap with the SPARE trial.

The blood samples will be sent to Viapath Analytics, the provider of pathology services at St Thomas’ Hospital, for testing with the 4 ELISA kits under investigation. All ELISA kits will be automated on Dynex DS2 2-Plate ELISA processing system in accordance with the manufacturer’s instructions for use. Sample analysis will be completed sequentially to avoid further freeze-thaw cycles. It is important to eliminate sources of variation that can be introduced due to repeated cycles. At the time point of analysis with each kit, all samples would have gone through the same treatment (ie, freeze-thaw cycles). Although measurement of uncertainty with each kit is known, drug/antidrug antibody complexes may show patient dependent variation upon freeze-thaw cycles and lead to further variation in different assay formats. This is accounted for by analysis of samples in identical integrity conditions. Pseudo-anonymized results from the testing will be compared with the results from the SPARE trial to validate the kits. Stored samples will be prepared, stored, and shipped according to the SPARE lab manual. Serum samples will be centrifuged and frozen before shipping. All biological samples will be retained within the central labs (in Israel) at –80°C for at least 6 years. All SPARE trial data will be collected in an electronic case report form by staff at participating sites. The Trial Statistician, Professor Sylvie Chevet (Biostatistics and Medical Information Department, Saint-Louis Hospital) will perform data collection and data quality controls.

### Statistical Analysis

All SPARE trial data are collected in an electronic case report form by staff at participating international sites. The data will be pseudo-anonymized. The Statistical Center in France will perform data collection and data quality control.

The coprimary outcomes are clinical relapse rate at 2 years and mean remission duration within 2 years. The following outcomes will also be assessed: (1) Inflammatory Bowel Disease Disability Index Scores, (2) Crohn Disease Activity Index, (3) adverse events, (4) trough levels of infliximab and fecal calprotectin, (5) direct and indirect costs associated with using or not using TDM, (6) health-related quality of life (EuroQol-5D and Short Health Scale), and (7) work productivity and activity index (Work Productivity and Activity Impairment Questionnaire in Crohn Disease).

### Ethics

All serum samples analyzed will be obtained during the SPARE trial. The SPARE trial was conducted following the principles of the International Conference on Harmonization Good Clinical Practice. A research ethics board reviewed the SPARE trial at the respective site. All subject information used in this study was deidentified for the subject identification number and investigational site. The informed consent process complied with the International Conference on Harmonization Good Clinical Practice and all applicable regulatory requirement(s). The consent of subjects included the use of the collected data and serum for other medical purposes. Therefore, additional consent for the current study was not required.

## Results

Funding has been sought to carry out this proposed research. This study is expected to take 18 months.

## Discussion

### Importance of the Study Results

The rationale behind conducting this study is to contribute to answering the questions identified by the research recommendations from DG22. If substantive evidence is generated, NICE will update its guidance to recommend the clinical use of one or more of the commercial ELISA kits for therapeutic monitoring and personalizing anti-TNF alpha inhibitor treatment. NICE guidance would encourage adoption both nationally and internationally, underpinning the potential value of this research. This technology has the potential to improve patient outcomes concerning clinical outcomes and patient-reported health-related quality of life. It also may be found to be cost effective. All of these are under investigation in our research.

Previous trials have found that varying infliximab in Crohn disease patients can have beneficial implications. One study, the “Stop Infliximab in Patients with Crohn's Disease” (STORI) trial (NCT00571337) [20] is an randomized controlled trial in France and Belgium which suggested that steroid-free remission may be maintained after infliximab discontinuation, with more than half of the 115 patients having reached sustained steroid-free remission after infliximab treatment with antimetabolites combination therapy for one to two years. This infliximab-free remission for a majority of the population will lead to substantial reductions in associated costs and side effects. This study also suggested that infliximab retreatment is safe and effective for relapsing patients. Further, in a 7-year follow-up, 21% of the population did not restart treatment with infliximab or another biologic [21]. However, the STORI trial did not have a control group of patients who were continuing infliximab treatment. Therefore, no results on when it is appropriate to recommend the withdrawal of infliximab from patients were discussed. The present study differs in that the population in focus is those continuing to use and respond to infliximab. Validating the technologies may lead to them being used clinically for informing treatment decisions and then, further clinical studies into the reduction or withdrawal of infliximab would be possible.

One advantage of utilizing the SPARE trial data for our research is that a quarter of the study population will be recruited from UK-based centers. This means that the cost data collected as part of the health economic analysis portion of our study will be directly relevant to treatment on the NHS. However, the circumstance of this study being an add-on to an international randomized controlled trial means we are highly dependent on the progress of that trial. Delays in the SPARE trial will impact on our progress and factors affecting recruitment numbers, or data quality will have a direct impact on our study. The samples obtained from the trial are to be retained for 6 years. Thus, it is highly likely we will be able to obtain the samples and conduct the validation study within this timeframe.

### Conclusion and Future Direction

The proposed study will validate 4 commercially available ELISA test kits and potentially impact on a NICE recommendation for using the technology clinically. This research is directly answering one of the research recommendations from NICE to investigate clinical outcomes associated with using the ELISA kits for TDM in people with Crohn disease who respond to treatment with TNF alpha inhibitors. Future studies into evaluating the clinical and cost effectiveness of the technologies prospectively in an NHS clinical environment will be a key aim for further research.

## Abbreviations

ADAL: adalimumab
ELISA: enzyme-linked immunosorbent assay
HRP: horseradish peroxidase
IFX: infliximab
IgG1: immunoglobulin G subclass 1
KiTEC: King’s Technology Evaluation Centre
NHS: National Health Service
NICE: National Institute for Health and Care Excellence
SONIC: Study of Biologic and Immunomodulator Naïve Patients in Crohn's Disease
SPARE: Prospective Randomized Controlled Trial Comparing Infliximab-Antimetabolites Combination Therapy to Antimetabolites Monotherapy and Infliximab Monotherapy in Crohn's Disease Patients in Sustained Steroid-Free Remission on Combination Therapy
STORI: Stop Infliximab in Patients with Crohn's Disease
TDM: therapeutic drug monitoring
TNF: tumor necrosis factor
UK: United Kingdom

## Appendix

Multimedia Appendix 1 A flowchart of the SPARE clinical trial.
