# Supplementary figures and images for: The Clinical and Cost-Effectiveness of 4 Enzyme-Linked Immunosorbent Assay Kits for Monitoring Infliximab in Crohn Disease Patients: Protocol for a Validation Study
Source: JMIR Res Protoc. 2018 Oct 19;7(10):e11218. doi: 10.2196/11218 (PMC6231806; doi:10.2196/11218)

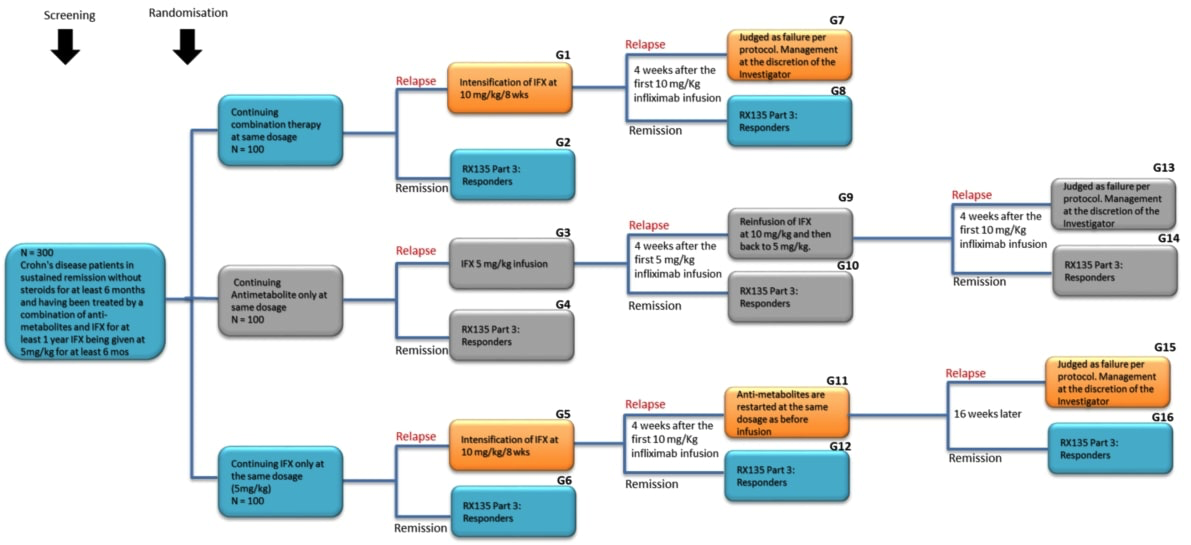

Supplement: Multimedia Appendix 1 [file 11218-218447-3-SP-png.png]
